# Müller's Muscle as a Sensory Proprioceptive Organ: Histological and Histochemical Analysis

**DOI:** 10.1167/iovs.64.5.18

**Published:** 2023-05-22

**Authors:** Daphna Landau-Prat, Chen Mayer, Nir Gomel, Mattan Arazi, Ofira Zloto, Amir Dori, Guy J. Ben Simon

**Affiliations:** 1 Goldschleger Eye Institute, Sheba Medical Center, Tel Hashomer, Israel; 2 The Talpiot Medical Leadership Program, Sheba Medical Center, Tel Hashomer, Israel; 3 Sackler Faculty of Medicine, Tel Aviv University, Tel Aviv, Israel; 4 Pathology Department, Sheba Medical Center, Tel Hashomer, Israel; 5 Department of Ophthalmology, Tel Aviv Sourasky Medical Center, Tel Aviv, Israel; 6 Neuromuscular Service, Sheba Medical Center, Tel Hashomer, Israel

**Keywords:** proprioception, ptosis, eyelid

## Abstract

**Purpose:**

The purpose of this study was to determine whether proprioceptive nerves are present in Müller's muscle.

**Methods:**

This was a prospective cohort study in which histologic and immunofluorescence analyses of excised Müller's muscle specimens were performed. Twenty fresh Müller's muscle's specimens from patients undergoing posterior approach ptosis surgery in one center between 2017 and 2018 were evaluated by histologic and immunofluorescent analysis. Axonal types were determined by measuring axon diameter in methylene blue stained plastic sections and by immunofluorescence of frozen sections.

**Results:**

We identified large (greater than 10 microns) and small myelinated fibers in the Müller's muscle, with 6.4% of these fibers being large. Immunofluorescent labeling with choline acetyltransferase showed no evidence of skeletal motor axons in the samples, indicating large axons are likely to be sensory and proprioceptive. In addition, we identified C-fibers using double labeling with peripherin and neural cell adhesion molecules.

**Conclusions:**

Overall, large myelinated sensory fibers are present in the Müller's muscle, likely serving proprioceptive innervation. This suggests that proprioception signals from Müller's muscle may have a role in eyelid spatial positioning and retracting, in addition to visual deprivation. This finding sheds new light on our understanding of this complex mechanism.

Upper eyelid retraction is believed to be maintained solely by contraction of the levator palpebrae superioris (LPS) and frontalis muscles, together with contraction of the sympathetically innervated Müller's muscle (MM). Several studies have suggested that the LPS also undergoes reflexive contractions, and that a visual stimulus may not be the only trigger for retractor muscle contractions.^1^^,^^2^ MM has also been hypothesized to contain proprioceptive neuronal structures, which elicit LPS muscle contraction via a stretch reflex served by the mesencephalic trigeminal nucleus. However, these studies did not distinguish between myelinated sensory proprioceptive and other large motor axons.^3^^,^^4^ Our aim was to identify proprioceptive structures in MM by means of histological examination and immunofluorescent labeling.

There is evidence, based largely on sural nerve studies, that nerve fiber function correlates with morphological classification.^5^ Accordingly, proprioceptive fibers may be identified by determining their size. Thus, we aimed to characterize and differentiate the various nerve fibers found in MM samples based on the size of the myelinated fibers. The nerve fibers classified as A-alpha are the largest (13–20 µm) of the myelinated axons. They belong to alpha motor neurons. Smaller large fibers (6–12 µm) are classified as A-beta axons, and have a sensory, proprioceptive function. Even smaller (1–5 µm) myelinated fibers are the A-delta fibers, and transmit sharp pain. C-type fibers are small (0.2–1.5 µm) and unmyelinated. They represent either pain/temperature sensory or sympathetic fibers. To address the study question, we looked for large, myelinated A-beta fibers in human MM samples. Although the MM is sympathetically innervated, we also aimed to rule out the presence of the other type of large, myelinated axons – the A-alpha fibers, which may be found in samples that include LPS, and that can be additionally excised in Müller's Muscle-Conjunctival Resection (MMCR) procedures. Myelinated fibers were demonstrated by anti-myelin basic protein (MBP) and/or anti-protein zero (P0) labeling, and unmyelinated fibers were demonstrated by their enclosure in neural cell adhesion molecule (NCAM)-labeled non-myelinated Schwann cells.^6^^,^^7^

## Methods

We included adult patients (>18 years) diagnosed with either unilateral or bilateral ptosis who underwent a posterior approach surgical correction (MMCR). The surgical procedures were performed by a single surgeon (author G.B.S.) at a single medical center between 2017 and 2018. The MMCR surgical approach involved the removal of the proximal part of the MM between the LPS and the tarsus of the upper eyelid. This excised tissue is generally not used, but is rather simply discarded. We obtained the patients’ consent to save these tissues, as well as carry out histologic examinations of the samples for the purposes of the current study. No alterations in the surgical procedure or in the postoperative patient management were needed. In addition to hematoxylin and eosin (H&E) staining, we used methylene blue staining and immunofluorescence. Immunofluorescence was used to identify myelinated versus unmyelinated fibers, and to rule out the presence of skeletal motor fibers. Methylene blue stained tissue was utilized for measuring axon diameter.

### Immunostaining

**Figure 1.**
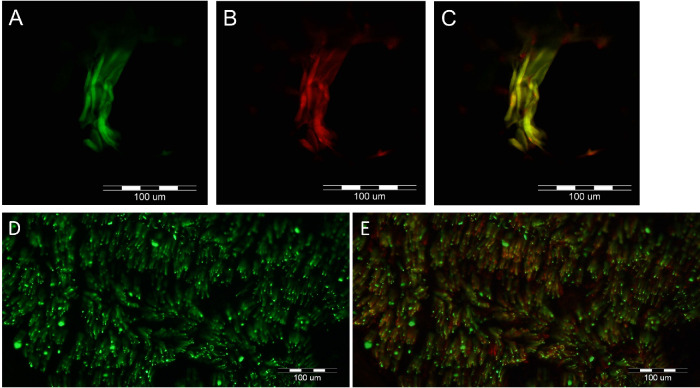
Deltoid muscle intramuscular motor nerves and sural nerve fascicle immunofluorescent labeling with anti-peripherin, anti-choline-acetyltransferase (ChAT), and anti-neural cell adhesion molecule (NCAM) immunoglobulins (Ig). These were used as positive controls to validate the immunoglobulins’ specificity. Anti-peripherin Ig stains all axons; anti-ChAT stains structures positive for ChAT, such as cholinergic neurons of motor nerves; and anti-NCAM antibodies stain non-myelinating Schwann cells surrounding unmyelinated axons. (**A–C**) Control labeling of deltoid muscle showing nerve fibers (anti-peripherin) green labeled axons that are also positive for choline-acetyltransferase (ChAT), indicating that they are motor nerve fibers. **A**, Anti-peripherin (*green*) immunoreactive nerve fibers. **B**, Anti-ChAT immunoreactivity (*red*). **C**, Merged view indicates double labeling of all the nerve fibers (*yellow-orange*). (**D,** **E**) Immunofluorescence of a cross-section of a sural nerve fascicule with anti-peripherin (*green*) in **D** to identify axons and with co-labeling of neural cell adhesion molecule (NCAM) which labels non-myelinating Schwann cells (*red*) in **E**. Unmyelinated sensory fibers are identified as yellow as they are labeled with both peripherin and NCAM, whereas myelinated fibers remain labeled green.

The samples were fresh-frozen, fixed in cold acetone, and cryo-sectioned at 12 microns. Sections were stained with H&E to identify the smooth muscle fibers of MM. Immunofluorescence was used to differentially identify axons, as well as myelinating and non-myelinating Schwann cells. Immunolabeling of MBP and/or P0 was used to identify myelin sheathes associated with myelinated axons.^8^ Schwann cells surrounding unmyelinated axons, that is, non-myelinating Schwann cells, were labeled with antibodies against the NCAM. Both myelinated and unmyelinated axons were labeled with anti-peripherin antibodies. Therefore, co-labeling of axons with peripherin and NCAM identified small unmyelinated fibers, classified as pain/temperature sensory C- or sympathetic fibers. Negative choline acetyltransferase (ChAT) labeling was used to rule out the presence of motor fibers. During MMCR procedures, due to the anatomic proximity of the MM to the LPS muscle, bundles of the LPS may be inadvertently included in the surgical specimen. Thus, when observing large myelinated axons, one has to ensure that these are not cranial nerve III fibers which innervate the LPS. The sural (pure sensory) nerve and intramuscular motor nerves in the deltoid muscle were used as positive controls (Fig. 1).

### Methylene Blue Processing

A separate set of samples were initially fixed in 2.5% glutaraldehyde in 0.1 M cacodylate buffer (pH 7.4) then post‐fixed in 1.0% OsO_4_ (EMS, USA) for 1 hour. Samples were then rinsed extensively in 0.1 M cacodylate buffer, dehydrated in a graded series of ethanols, and embedded in Epoxy resin (Agar Scientific, United Kingdom). The embedded samples were cured at 60°C. Then, 1.5 µm sections were cut using a Leica Ultracut UC7 ultramicrotome (Leica Microsystems Inc., LKB‐II, Germany). These were stained with a 1% solution of methylene blue (Sigma, Germany). Following slide preparation, the slides were then digitally scanned using whole slide imaging and the diameters of myelinated axons were measured, with required measurements taken digitally. All measurements were accurately performed by a single dedicated trained pathologist (author C.M.) using the Phillips slide viewer (Image Management System 3.3.1 Phillips IntelliSite Pathology Solution) and were not conducted automatically. The measured diameters included the axon and its myelin sheath. The measurements were performed on the scanned slides while simultaneously observing the actual slides with the microscope to include all areas, and to focus through the material. The magnification was times 40 to 100. In cases of seemingly elongated fibers, which may represent oblique cuts through more circular nerves, the diameter measurement was performed over the shortest axis rather than the largest one.

### Collaboration and Ethics

This interdisciplinary study was conducted in collaboration with the Ophthalmology Department, Pathology Department, and the Neuromuscular Pathology Unit at Sheba Medical Center. It was approved by the local institutional review board (IRB), and informed consent was given by all participants.

## Results

**Figure 2.**
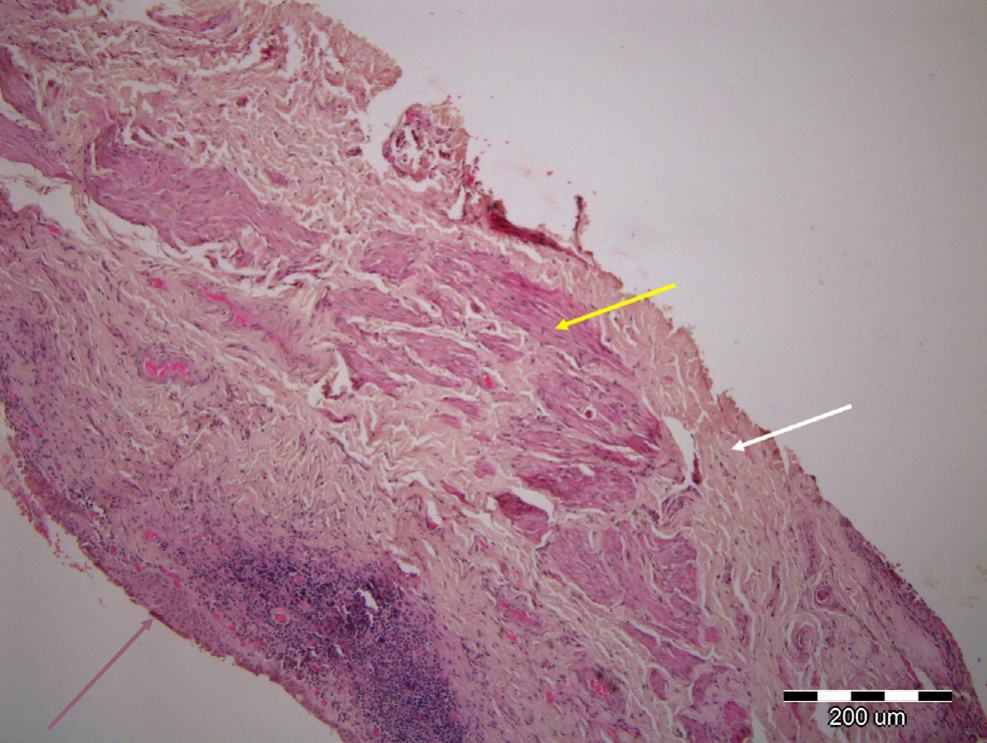
Hematoxylin and eosin (H&E) staining of the specimens taken from Müller's muscle conjunctival resection procedure, showing smooth muscle cells (*yellow arrow*) surrounded by connective tissue (*white arrow*) and lined by epithelial conjunctival cells (*pink arrow*).

Twenty MMCR specimens were obtained from 12 patients (8 patients had bilateral surgery), comprising 5 female patients and 7 male patients, mean age of 62 years (range = 38–77 years). H&E staining of the specimens showed smooth muscle cells surrounded by connective tissue and lined by epithelial conjunctival cells (Fig. 2). MM fibers were noted in all specimens prior to immunostaining with H&E.

### Immunohistochemical Staining and Fluorescence Microscopy

**Figure 3.**
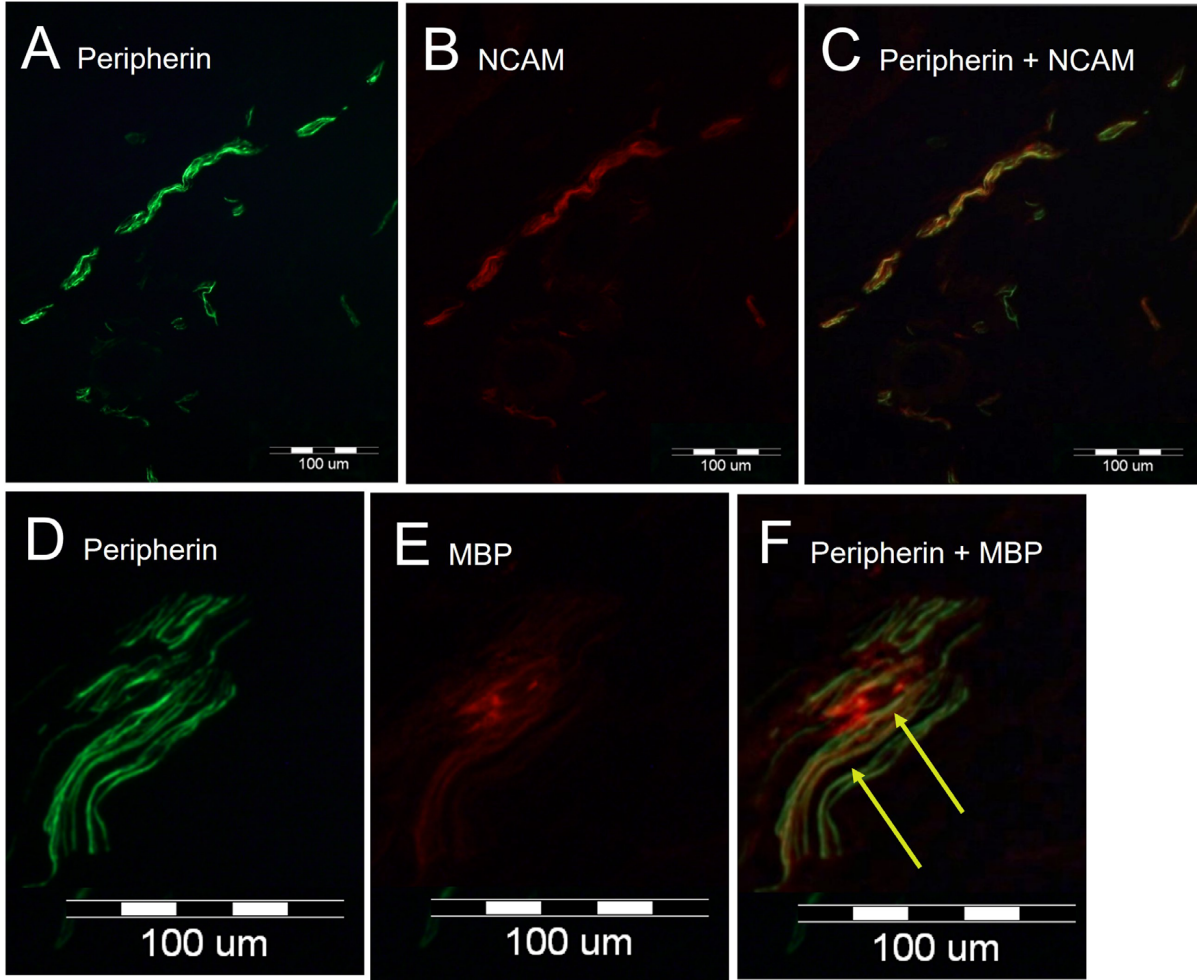
Immunofluorescent microscopy labeling of a Müller's muscle (MM) specimen, which demonstrates both myelinated and unmyelinated fibers in the MM. (**A–C**) Anti-peripherin (**A**, *green*) Ig stains all axons; anti-neural cell adhesion molecule (NCAM) Ig (**B**, *red*) stains non-myelinating Schwann cells. Double labeling demonstrates the presence of unmyelinated axons (**C**, *yellow* in merged image). (**D,** **E**) Anti-peripherin (**D**, *green*) labels all axons; anti-myelin basic protein (**E**, *red*) labels myelinating Schwann cells. Double labeling denotes the presence of myelinated axons (**F**, *arrow*, *yellow* in merged image).

Various axon group types were represented in all MM specimens (Fig. 3). The first group consisted of thin axons, which were double stained for both peripherin and NCAMs, denoting thin unmyelinated axonal fibers (see Figs. 3A–C). The second group consisted of thin axons, which were double labeled (yellow arrow) for both peripherin and MBP/P0 antibodies, denoting thin myelinated axons (see Figs. 3D–F).

**Figure 4.**
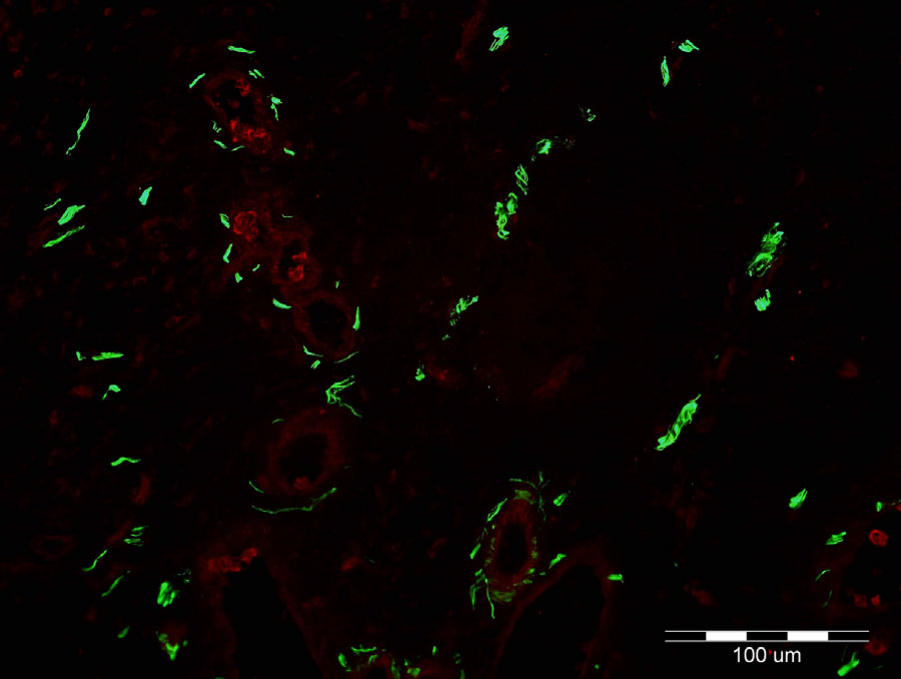
Immunofluorescent microscopy demonstrates labeling of Müller muscle (MM) with anti-peripherin (*green*) and anti-choline-acetyltransferase (ChAT) (*red*) immunoglobulins. Anti-peripherin Ig labels all axons; anti-ChAT labels structures positive for ChAT, such as skeletal motor nerves. Green structures represent positive anti-perpherin immunolabeling of axons (*green*). Note weak ChAT staining in the muscle fibers. None of the axons in this sample, as well as all other samples, were positive for ChAT, ruling out the presence of motor axons.

Anti-peripherin axonal labeling denoted similar alignment of most fibers. None of the peripherin-positive fibers were ChAT-positive, even though weak ChAT-positive staining was present in the muscle fibers (Fig. 4). Presumably, the peripherin-positive axons co-labeled for NCAM represent sensory fibers conveying pain and temperature information, along with postganglionic sympathetic fibers supplying MM, whereas the peripherin-positive axons co-labeled by MBP likely include both A delta (sharp pain) and A beta (proprioceptive) fibers; the presence of motor fibers was eliminated by the negative ChAT axonal staining.

### Methylene Blue Staining

**Figure 5.**
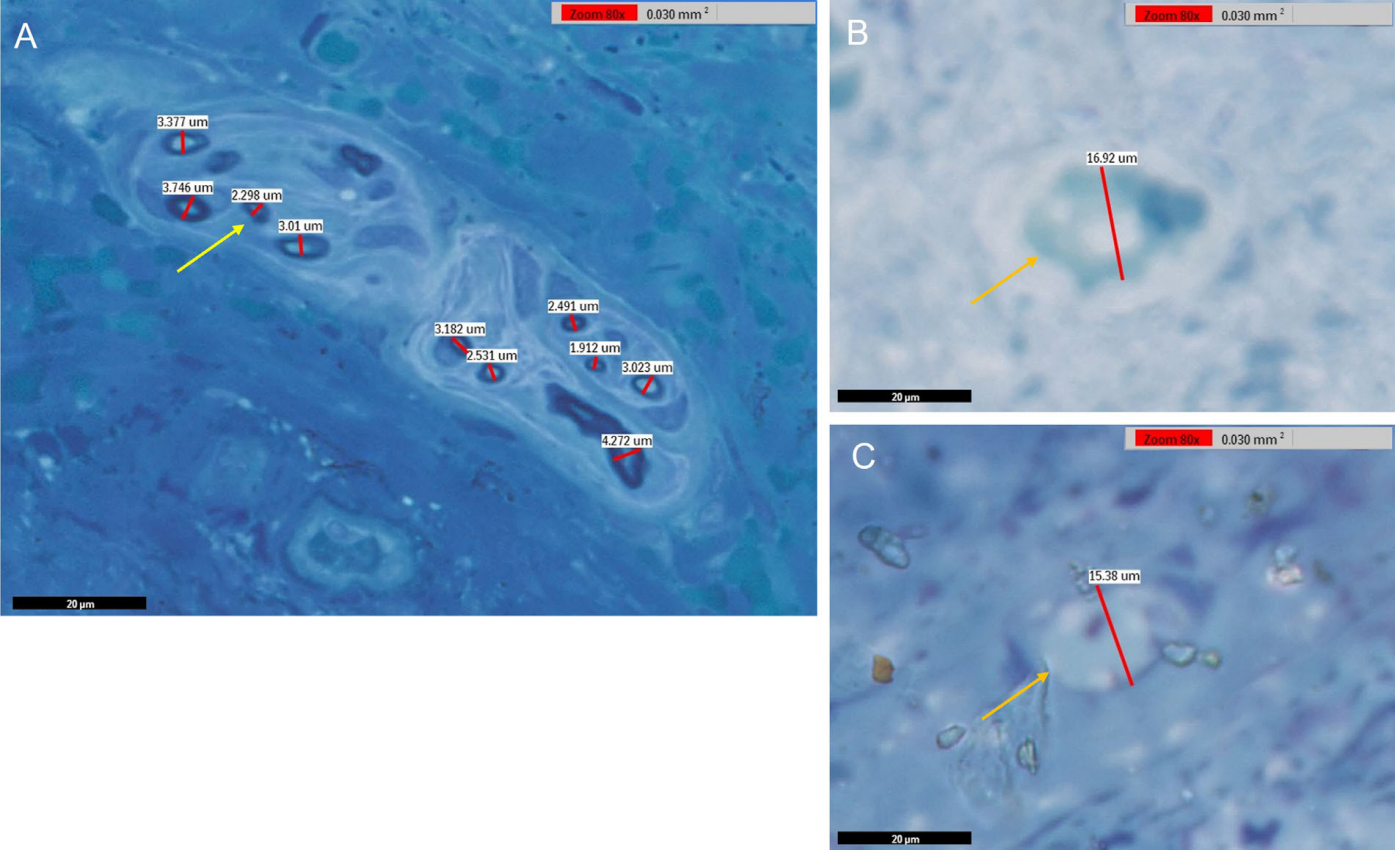
Scanned slides depicting cut sections of fixed Müller muscles stained with methylene blue. (**A**) Cross-section of a nerve bundle between muscle fibers. (**B,** **C**) Cross-sections of myelinated nerves with larger diameters (**B**, 16.92 µm, **C**, 15.38 µm). As highlighted by the stain, individual axons are encased in myelin. The *arrows* indicate axons of various sizes (*yellow* = smaller diameter and *orange* = larger diameter). In cases of seemingly elongated fibers, which may represent oblique cuts of circular axon profiles, the diameter measurement was performed over the shortest axis rather than the long one; using this rule, the true diameter was obtained. Overall, 580 usable axons were located and measured, with an average diameter of 5.37 micrometers (standard deviation = 2.89).

**Figure 6.**
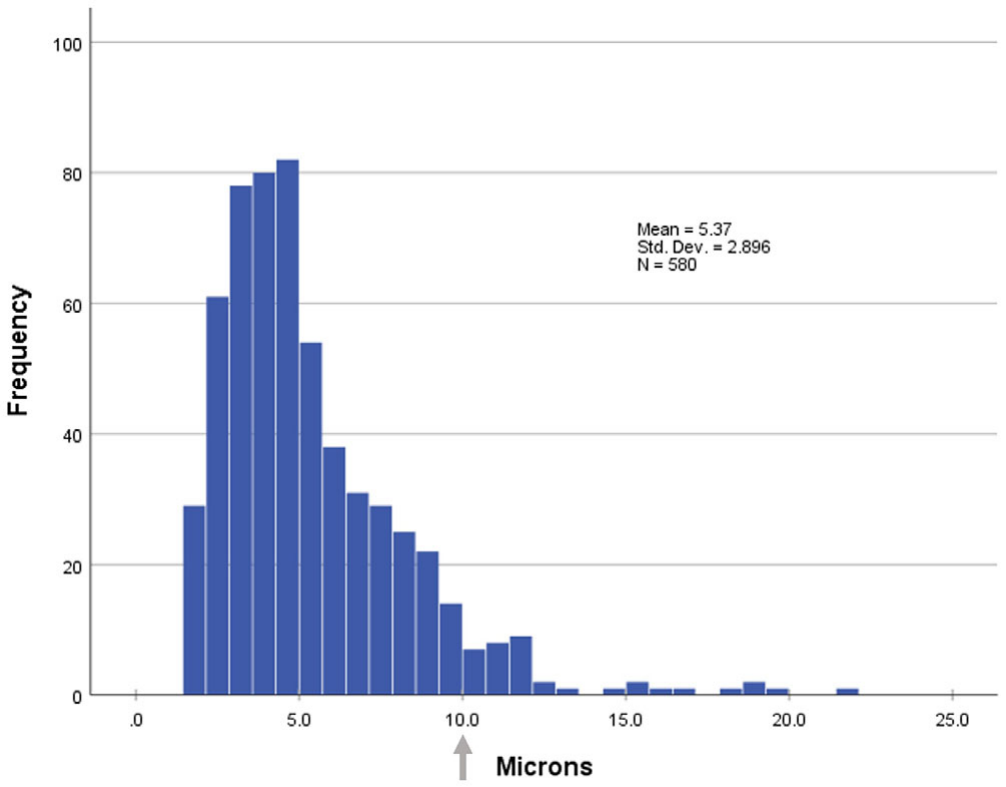
Diameter distribution of 580 myelinated axons observed in Müller's muscle samples. The average diameter was 5.37 micrometers (standard deviation = 2.89); 37 axons (6.4%) measured above 10 micrometers (arrow), with the largest being 21.68 micrometer in diameter. The larger axons correspond to A-beta fibers, indicating that they are proprioceptive.

We obtained 4 different MM tissue samples, which were separated into 16 tissue blocks. These were later sectioned and stained, as detailed in the Methods section. We then digitally reviewed these slides in search of cross-sections through myelinated nerve fibers (Fig. 5). Out of 16 scanned slides, 580 fibers were located and measured, with an average fiber diameter of 5.37 micrometers (standard deviation = 2.89). Of these fibers, 37 fibers (6.4%) measured greater than 10 micrometers, the cutoff point for A-beta axons, with the largest being 21.68 micrometers in diameter. The axons’ distribution is shown in the histogram in Figure 6. Of note, a bimodal distribution was not observed. These larger axons might be either A-beta proprioceptors or they might be skeletal motor fibers supplying LPS. However, as none of the peripherin-positive fibers were ChAT-positive, this strongly indicates that this group of thicker axons represent proprioceptive, A-beta sensory fibers.

## Discussion

In the current study, immunohistochemistry demonstrated the presence of both myelinated and non-myelinated nerve fibers in the MM of patients undergoing posterior approach ptosis surgery. Methylene blue staining enabled measurements of these myelinated fibers, and proved the presence of both thick and thin fibers. None of these fibers stained for ChAT, hence they were most likely sensory nerves or postganglionic sympathetic axons. In this series, 6.4% of the myelinated fibers had a diameter of over 10 microns, supporting the hypothesis that these thicker myelinated sensory fibers are proprioceptive, unlike the abundant thin fibers, which are more compatible with common pain/temperature sensory C-fibers or sympathetic fibers.

Eyelid position is maintained by a delicate balance between eyelid retractors comprised of the LPS muscle, MM, and the frontalis muscle, as well as the eyelid protractors, which are comprised mainly of the orbicularis oculi muscle.^9^^–^^11^ Evinger et al. have presented strong evidence that lid position does not involve the orbicularis oculi muscle when the eyes are open.^12^ LPS neural signaling is also influenced by vertical eye movements, as well as the blink reflex, which are required for coordinated lid and eye movements.^13^^–^^15^ Visual disturbance, mainly by loss of the upper field due to eyelid drop, was believed to be the main trigger for eyelid retraction. However, several reports have suggested that additional factors may also influence muscle activation, as observed in patients with poor or no vision.^1^^,^^16^^,^^17^ Another theory worthy of consideration is whether the sympathetic input to MM actually regulates the degree of activation of the proprioceptive feedback to the LPS muscle. If so, when one tires, decreased autonomic tone would lead to decreased activity to the LPS, closing the eye more completely than just the sympathetic drive loss would.

The complex relations between eyelid and eyebrow position remains to be elucidated. We and others^6^ have shown that patients with long-standing eyelid ptosis may paradoxically continue utilizing the frontalis muscle after successful surgical correction, despite good postoperative eyelid position. This suggests that a visual stimulus may not be the only trigger for compensatory frontalis muscle contraction in patients with ptosis. Many patients with long-standing upper eyelid ptosis use their frontalis muscles as a compensatory mechanism for their contracted visual field.^16^ However, patients with anophthalmia may also exhibit frontalis activation in ptotic eyelids, suggesting that a contracted visual field cannot be the sole stimulus for compensatory brow elevation.^18^^,^^19^ A sensory or proprioceptive mechanism for frontalis function in relation to eyelid position has been hypothesized.^18^^,^^19^ In addition, patients with enophthalmos may demonstrate frontalis recruitment. We suggest that posterior displacement of MM and/or the LPS may trigger brow elevation.

Several studies have demonstrated the complexity of the relationship between MMCR and the amount of ptosis correction. Histological examination of MM specimens has demonstrated that there is no correlation between the amount of a resected MM and the extent of postoperative eyelid elevation.^2^ In addition, pharmacologic investigations of adrenergic receptors in MM failed to find any predictable association between the number of receptors and the tissue response, or between tissue response and the surgical outcome, suggesting that mechanical and/or pharmacologic factors related to MM play only minor or indirect roles in the mechanism of MMCR.^20^^,^^21^

Various surgical approaches for tissue removal in MMCR result in similar outcomes. Rootman et al.^1^ compared the use of a standard 7 mm resection length to a variable 4:1 ratio of resection length to achieve the desired elevation nomogram when performing MMCR. These authors found no significant postoperative differences in eyelid position between the two groups, and concluded that these results tend to argue against the success of MMCR surgery being the result of a mechanical correction alone. They also concluded that there are complex relations between the muscles responsible for eyelid position and a more comprehensive eyelid position maintenance system, which includes afferent and efferent pathways and central processing.

Taken together, the results of the above studies support the conclusion that the function of MM and its relation to the other eyelid retractors have yet to be fully understood. These observations further suggest that eyelid spatial position, and not visual deprivation alone, affects the activation of eyelid retractors, thus implying the existence of a proprioceptive mechanism in the eyelid.

### Proprioceptive Mechanism in the Eyelid

Several studies hypothesized that MM contains mechanoreceptors and proprioceptive neuronal structures, which elicit LPS and frontalis contractions via the mesencephalic trigeminal nucleus in the form of excitatory proprioceptive feedback indicating the position of the upper eyelid.^3^^,^^4^^,^^22^^,^^23^ Matsuo et al.^23^ demonstrated that intra-operative stretching of a unilateral MM evoked bilateral contraction of the LPS. Of note, there is good evidence that the innervation of the levator is from motoneurons on both sides of the caudal central subdivision of cranial nerve III, with some motoneurons even projecting bilaterally.^24^^–^^26^ Ban et al.^22^ reported that unilateral electrical stimulation of the transverse trigeminal proprioceptive nerve proximal to MM during surgery under general anesthesia induced a short latency response in the ipsilateral LPS muscle in the form of a trigemino-oculomotor withdrawal reflex. Others showed that unilateral transcutaneous electrical stimulation of the trigeminal proprioceptive fibers that innervate the mechanoreceptors in MM induce electromyographic responses in the frontalis muscles in the form of a trigeminofacial reflex.^27^

Animal histologic studies have confirmed the presence of Golgi tendon organs, muscle spindles, and myotendinous cylinders within the extraocular muscles,^28^ including the LPS of domestic sheep and the moufflon, a wild sheep.^29^^,^^30^ These specialized structures can detect LPS stretch, and additionally stimulate ipsilateral frontalis recruitment. Fujita et al.^31^ have claimed that cell bodies of the trigeminal proprioceptive neurons, that stimulate reflexive contraction of the LPS muscle, are located in the mesencephalic trigeminal nucleus in rats. Human histologic studies have also yielded supporting evidence of eyelid proprioception. Yuzuriha et al.^3^^,^^4^ found fine neural myelinated structures in MMs of human cadaver eyelids and claimed that these structures act as mechanoreceptors. They concluded that MM is innervated by the unmyelinated sympathetic efferent fibers, as well as by the myelinated trigeminal proprioceptive afferent fibers, both of which run transversely on the proximal MM to join the lacrimal nerve. Yuzuriha et al.^32^ stained interstitial cells of Cajal among MM fibers, and suggested that they may serve as mechanoreceptors by both contacting MM fibers and forming associations with trigeminal proprioceptive fibers to induce reflexive contraction of the LPS and frontalis muscles. Vrcek at el.^33^ analyzed MM and LPS samples using anti-synaptophysin to label synaptic vesicles in nerve terminals, anti-neurofilament IgG to label nerve fibers, phalloidin to label muscle fibers, and α-bungarotoxin to label motor terminals. They described synaptophysin-positive free nerve terminals within the intermuscular connective tissue of MM. This is anatomically consistent with free nerve endings found in the extraocular muscles that have been implicated in proprioception, although their function is still debated.^34^^–^^37^ The success of MMCR may be attributed to alterations in the proprioceptive structures, with a resultant change in LPS activity, as well as mechanical correction by means of posterior lamella (conjunctiva and MM) shortening and LPS advancement. Proprioception is suggested as an additional mechanism to maintain eyelid position above the pupillary margin, and to induce eyelid elevation in cases of ptosis. In this surgery, we do not remove the entire MM. Moreover, in failed cases, a repeated procedure commonly helps. Hence, it is possible that the decreased total number of remaining proprioceptive fibers and/or their altered spatial distribution within the postoperative posterior lamella tissue can influence eyelid elevation; the direction of this influence, however, remains unknown. Of note, in some cases, the primary postoperative eyelid position may be the same or even lower than the pre-operative position, even after the edema resolves, whereas the final outcome seen a few weeks later is significantly improved. This may imply a dynamic mechanism, rather than a purely mechanical surgical effect, with a possible “reset” of the neuronal mechanism taking place. Future studies can investigate the presence of remaining fibers in repeated surgeries, and explore whether their amount or anatomic distribution is associated with surgical success.

A thorough understanding of the mechanisms underlying eyelid function is especially important in the management of children with ptosis who may have co-existing amblyopia. Visual stimulation had been believed to be the sole trigger of eyelid retraction. Children with mild ptosis that does not obstruct the visual axis with frontalis recruitment are sometimes managed conservatively under the assumption that no suppression occurs if the eyelid retractors are recruited. However, understanding that eyelid position may activate the LPS and frontalis muscles may stimulate re-thinking of past management, as well as lead to further considerations regarding implementation of early surgical solutions or more aggressive amblyopia treatment, especially in cases where the eyelid margin is positioned at or above the upper border of the pupil. Such interventions would be intended to prevent deprivation or anisometropic amblyopia.

Limitations of this study stem from the fact that the cut off sizes used here are based on previously reported values in other muscles. No bimodal distribution was observed to distinguish between small and large myelinated fibers. Another limitation is that the presence of encapsulated proprioceptors (Golgi tendon organs and neuromuscular spindles) was not investigated in this study, as these are found in skeletal muscles and were not expected to be found in our specimens, which mainly contain smooth muscles fibers of MM.^38^ In addition, anti-SMA immunostaining to reveal smooth muscle tissue was not performed.

Although the cumulative results of these studies support the theoretical existence of a proprioception mechanism in MM, few studies have thus far provided actual pathologic or any other evidence to verify the presence of any proprioceptive anatomical structures in MM.^20^ Our findings may, therefore, provide supportive anatomic evidence for a proprioceptive mechanism in the eyelid. Its presence can explain why eyelid spatial position, and not visual deprivation alone, stimulate retractor activation. This raises the possibility that mechanical manipulation of a motor system may allow for sensory processing centers to recalibrate and maintain eyelid position. Further studies are warranted in order to continue exploring this complex mechanism that plays a major role in periocular function.
